# Missed transplanting rate evaluation method for tobacco seedling transplanter based on UAV imagery and improved YOLOv5s

**DOI:** 10.3389/fpls.2025.1659559

**Published:** 2025-09-24

**Authors:** Rui Su, Bei Yu, Yufei Sun, Ling Wang, Lei Gao, Du Chen

**Affiliations:** ^1^ College of Engineering, China Agricultural University, Beijing, China; ^2^ Hunan Tobacco Research Institute, Changsha, Hunan, China; ^3^ State Key Laboratory of Intelligent Agricultural Power Equipment, China Agricultural University, Beijing, China

**Keywords:** missed transplanting rate, UAV imagery, deep learning, seedling detection, target counting

## Abstract

Missed transplanting remains a significant challenge in the application of tobacco seedling transplanters due to the specific agronomic requirements for successful transplanting. Currently, the detection of missed transplanting rate in large-scale field tests primarily relies on manual seedling counting, a process that is notably inefficient. Traditional online detection methods, including photoelectric sensors and machine vision, suffer from problems such as complex structures and high costs. They require sensor deployment on the machine itself, making it difficult to fully meet the actual detection needs of transplanters during the R&D and testing phase. To address these limitations, this paper proposes an automated evaluation method for detecting missed transplanting rates using UAV (unmanned aerial vehicle) imagery. The method integrates an improved YOLOv5s model, DeepSORT, and line-crossing counting approach. First, a second-order channel attention (SOCA) attention mechanism was incorporated into the YOLOv5s model to improve its ability to extract features for small targets. Additionally, the Spatial Pyramid Pooling Fast (SPPF) was replaced by the Simplified Spatial Pyramid Pooling-Fast (SimSPPF) to enhance the model’s ability to extract multi-scale features for targets such as seedling-planted holes. The DeepSORT algorithm, combined with line-crossing counting principle, was then employed for visual tracking and dynamic counting of seedling-planted and missed-planting holes, enabling accurate evaluation of the missed transplanting rate. Test results showed that, in terms of target detection, the Precision and mAP of the improved YOLOv5s model increased by 3.9% and 5.3%, respectively, compared to the original YOLOv5s. In target tracking, the combination of the improved YOLOv5s and DeepSORT reduced the missed detection rate *M_m_* and false detection rate *M_f_* by 2.5% and 6.1%, respectively. Field experiments achieved an accuracy of 90.28% for the missed transplanting rate and a 10× higher detection efficiency compared to manual inspection. This method offers a novel automated solution for the rapid detection of missed transplanting rates in large-scale transplanting operations and provides valuable technical insights for evaluating the performance of other seedling transplanters.

## 1 Introduction

Tobacco is a significant special economic crop, serving as a major source of national fiscal revenue and playing a vital role in the global agricultural economy. The hilly and mountainous regions of Southwest China are the primary tobacco-growing areas, contributing approximately 62.53% of the nation’s total tobacco production (Tong et al., 2020). These regions, characterized by complex terrain and poor soil quality, limit the economic viability of traditional grain crops. In contrast, tobacco, as a high-value crop, enhances land-use efficiency and economic returns, providing a crucial means for local farmers to increase income and achieve prosperity (Li et al., 2023a). Reducing production costs and labor input, thereby lowering expenses and boosting efficiency, is essential for increasing farmers’ income and advancing industrial upgrades (Tarolli and Straffelini, 2020).

Hilly and mountainous areas currently face delayed development in agricultural mechanization, with tobacco production still heavily reliant on traditional manual labor. This dependence makes it a typical labor-intensive industry characterized by high production intensity and low efficiency. Labor shortages and rising labor costs are further rendering the traditional production model inadequate to meet the demands of modern agricultural development (Tarolli and Straffelini, 2020). Consequently, promoting mechanization in tobacco fields has become an inevitable trend in advancing modern tobacco agriculture. However, in hilly and mountainous regions, steep slopes and fragmented arable land present substantial challenges. The existing level of mechanization equipment remains inadequate to meet the specific demands of the tobacco industry in these areas (Wu et al., 2022). Therefore, accelerating improvements in mechanization and promoting the adoption of relevant technologies are crucial for enhancing tobacco production in these regions.

Currently, tobacco production remains highly reliant on traditional manual labor, characterized by high labor intensity and low efficiency. In the full mechanization of tobacco production, the mechanization rate of soil tillage and preparation is relatively high, while the “planting” and “harvesting” stages are still quite underdeveloped. Taking the “planting” stage as an example, tobacco cultivation involves specific agronomic requirements. For instance, the well-cellar transplanting technology demands that the machine not only achieves high-speed cellar digging but also maintains a high degree of coordination with the seedling feeding device (Li et al., 2023; Lin et al., 2023). This poses significant challenges to tobacco seedling transplanters at the current stage, which is why domestic tobacco seedling transplanters are still in the research, development, and testing phase. The rapid evaluation of key performance indicators of tobacco seedling transplanters during research and testing can accelerate the identification and optimization of machine-related issues, improve testing efficiency, ensure transplanting quality, and promote standardized detection (Xu et al., 2022).

Traditional methods for evaluating transplanter operation success rate primarily rely on manual counting, which suffers from low efficiency and high labor intensity (Lin et al., 2023b). For large-scale operations, evaluations often involve either rough qualitative assessments based on casual observation or random sampling, making it challenging to accurately quantify the key performance indicators of seedling transplanters during extensive transplanting tests. Researchers have extensively studied online detection methods for operation performance, employing technologies such as photoelectric sensors and cameras. (Jin et al., 2018) developed an intelligent transplanting system using photoelectric sensors to bypass empty soil bases in potted chili seedlings, enabling automatic recognition of seedlings under indoor transplanting conditions. (Jiang et al., 2022) proposed a real-time detection method for missed transplanting based on video image stitching, achieving 92.32% recognition accuracy in monitoring missed transplanting of rapeseed blanket-type seedlings during field operations. However, the effectiveness of these online detection methods is often influenced by the agricultural background. Additionally, the high cost of these sensors makes them unsuitable for seedling transplanters still in the R&D stage, where structural designs are not yet finalized (Wu et al., 2025). Therefore, to address these limitations, an operation performance evaluation method independent of the transplanter’s design is needed to better meet the practical requirements of current machine testing. An automatic transplanting detection system, which combines UAV images and machine vision technology, offers an effective solution to this challenge (Ji et al., 2020; Huang et al., 2024).

The key to evaluating the operational performance of transplanters lies in the accurate detection of various target states of seedlings after transplanting. Currently, the integration of drones and machine vision has been widely adopted for crop seedling monitoring. Early machine vision-based object detection algorithms typically rely on shallow features such as crop color, shape, and texture, employing morphological operations for image segmentation to identify target regions. (Zhao et al., 2017) performed target recognition and segmentation of rapeseed plants based on the color vegetation index and Otsu threshold algorithm and estimated the number of rapeseed plants through remote sensing image features. but remote sensing images are greatly affected by weather conditions. (Xie et al., 2016) utilized support vector machines (SVM) to identify and count tobacco plants from drone-acquired images in the Lab color space, achieving a 96.1% accuracy rate. However, these methods are susceptible to factors such as terrain and illumination. Moreover, they require manual input of features and adjustment of feature thresholds, making it difficult to ensure the recognition performance in complex environments (Huang et al., 2024).

With the continuous advancement of deep learning technology and improvements in computer hardware performance, deep learning-based crop counting methods are gradually replacing traditional machine vision techniques. These methods have been successfully applied to the detection of crop targets such as wheat spikes (Bao et al., 2023), tobacco (Rao et al., 2023), and strawberries (Li et al., 2023c), achieving the desired detection accuracy. (Barreto et al., 2021) utilized Fully Convolutional Networks (FCNs) to segment seedling phenotypes, enabling fully automated plant counting in sugar beet, corn, and strawberry fields. Similarly, (Machefer et al., 2020) applied the Mask R-CNN network to count two low-density crops, potatoes and lettuce. For object detection-based methods, (Bao et al., 2023) developed a wheat ear detection model using TPH-YOLO (YOLO with transformer prediction heads) to count wheat ears from drone-acquired images, achieving an accuracy of 87.2%. Despite the widespread application of static image-based counting methods in agriculture, challenges persist. Low-quality UAV images, along with stitching errors and overlaps during the stitching process, significantly impact the accuracy of crop target detection and counting (Cui et al., 2023).

The effectiveness of video stream-based crop counting methods relies on accurately identifying and associating the same target across multiple frames, with tracking algorithms being among the most widely used approaches. In recent years, dynamic tracking methods such as SORT (Bewley et al., 2016) and DeepSORT (Veeramani et al., 2018) have been widely applied in various agricultural domains. These methods integrate Kalman filters with the Hungarian algorithm to perform multi-object tracking and dynamic counting in diverse environments while maintaining high counting accuracy. For instance, (Lin et al., 2022) integrated the YOLOv5s object detection model with the DeepSORT tracking algorithm to develop an efficient low-altitude real-time peanut counting model, achieving an accuracy of 98.08%. Similarly, (Wang et al., 2021) employed the YOLOv3 network with Kalman filters to create a maize seedling counting method, achieving an accuracy exceeding 98%. Compared to static image-based crop counting methods, video-based approaches offer significant advantages in detection efficiency. However, the accuracy of counting results heavily depends on the performance of both the detection model and the tracking algorithm. Thus, developing efficient detection models and tracking algorithms is crucial for ensuring accurate crop counting in video streams (Cui et al., 2023).

Although deep learning-based object counting methods have been widely applied, directly utilizing existing methods from the literature to evaluate the performance of special crop seedling transplanting operations presents several challenges. First, most crop detection studies focus primarily on seedling counting and rarely consider the identification of missed transplanting. Second, the unique transplanting environment and agronomic characteristics of tobacco seedlings, such as the Well-Type transplanting technique, result in seedlings being entirely enclosed within transplanted holes after planting. This leaves only limited pixel points visible in high-altitude drone imagery, making it difficult for detection models to differentiate between planted and missed seedlings. Additionally, background variations, including bare soil, white plastic film, and green weeds, significantly hampers model performance in real-world applications.

To overcome the limitations of previous studies, this paper proposes an automatic transplanting quality assessment system, utilizing UAV imagery and an improved YOLOv5s model. The detection process for the method proposed is as follows: First, a large-scale high-altitude dataset of tobacco mechanized transplanting operations is created using drone-acquired images. Then, a detection network based on the improved YOLOv5s model is trained and optimized to achieve the required accuracy. Building upon this network, the DeepSORT tracking algorithm and line-crossing counting method are integrated to develop an automated performance evaluation model for tobacco seedling transplanters. This model allows accurate detection of the missed transplanting rate, an operational indicator of transplanters, during large-scale transplanting operations.

## 2 Materials and methods

### 2.1 Overview of methods

Through field tests and investigations, we found that the missed transplanting rate remains a critical factor limiting the widespread adoption of tobacco seedling transplanters. Accordingly, this study focuses on the precise detection of missed transplanting rates in seedling transplanters. Specifically, missed planting in tobacco transplanting is defined as locations where tobacco seedlings should theoretically be planted but are actually not. Meanwhile, the missed transplanting rate is defined as the ratio of the total number of missed-planting holes to the total number of planted holes, calculated using Equation 1.


(1) $$Missed\;transplanting\;Rate=\frac{T_{n}}{T_{N}}\times 100\%$$

Where *T_n_* represents the total number of missed-planting holes, and *T_N_* denotes the total number of planted holes measured by the tobacco seedling transplanter. The total number of planted holes includes both seedling-planted and missed-planting holes. Therefore, the key to accurately determining the missed transplanting rate lies in effectively distinguishing and identifying seedling-planted holes from missed-planting holes.

**Figure 1:**
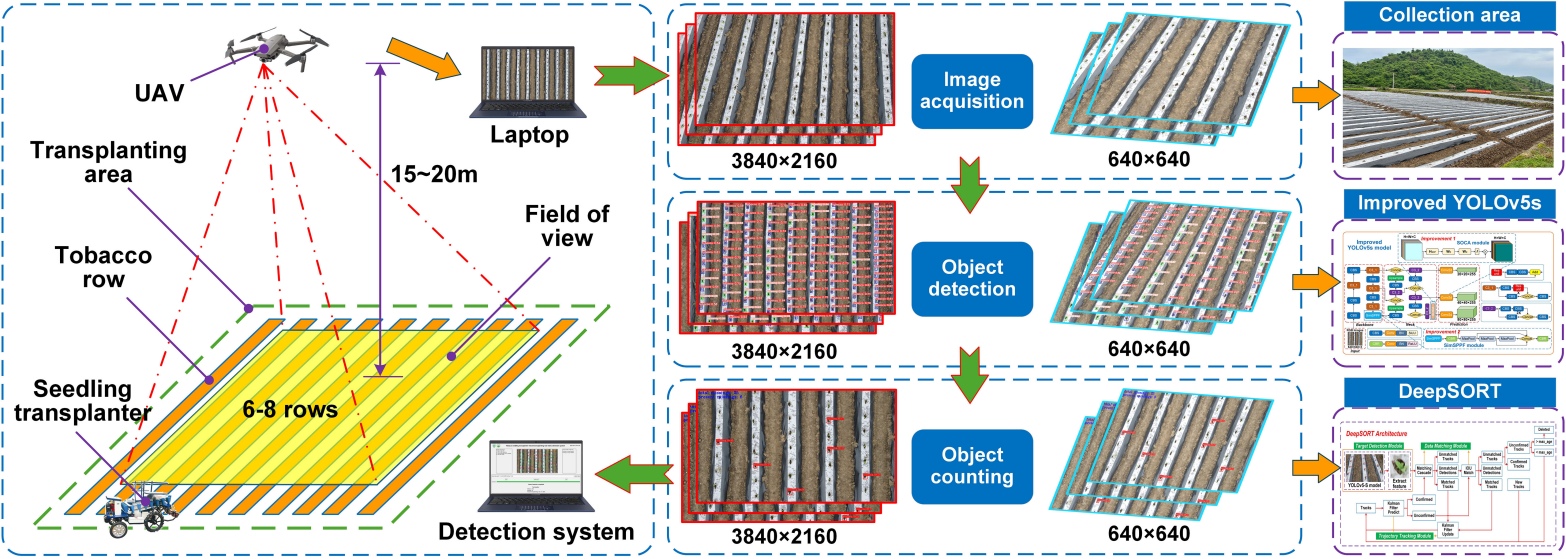
Technical solution for dynamic counting.

This study aims to develop an automated detection system for the missed transplanting rate of tobacco seedling transplanters based on the improved YOLOv5s and DeepSORT algorithms. **Figure 1** illustrates the overall technical workflow of the proposed method, with detailed descriptions of each step provided below.

### 2.2 Dataset construction

**Figure 2:**
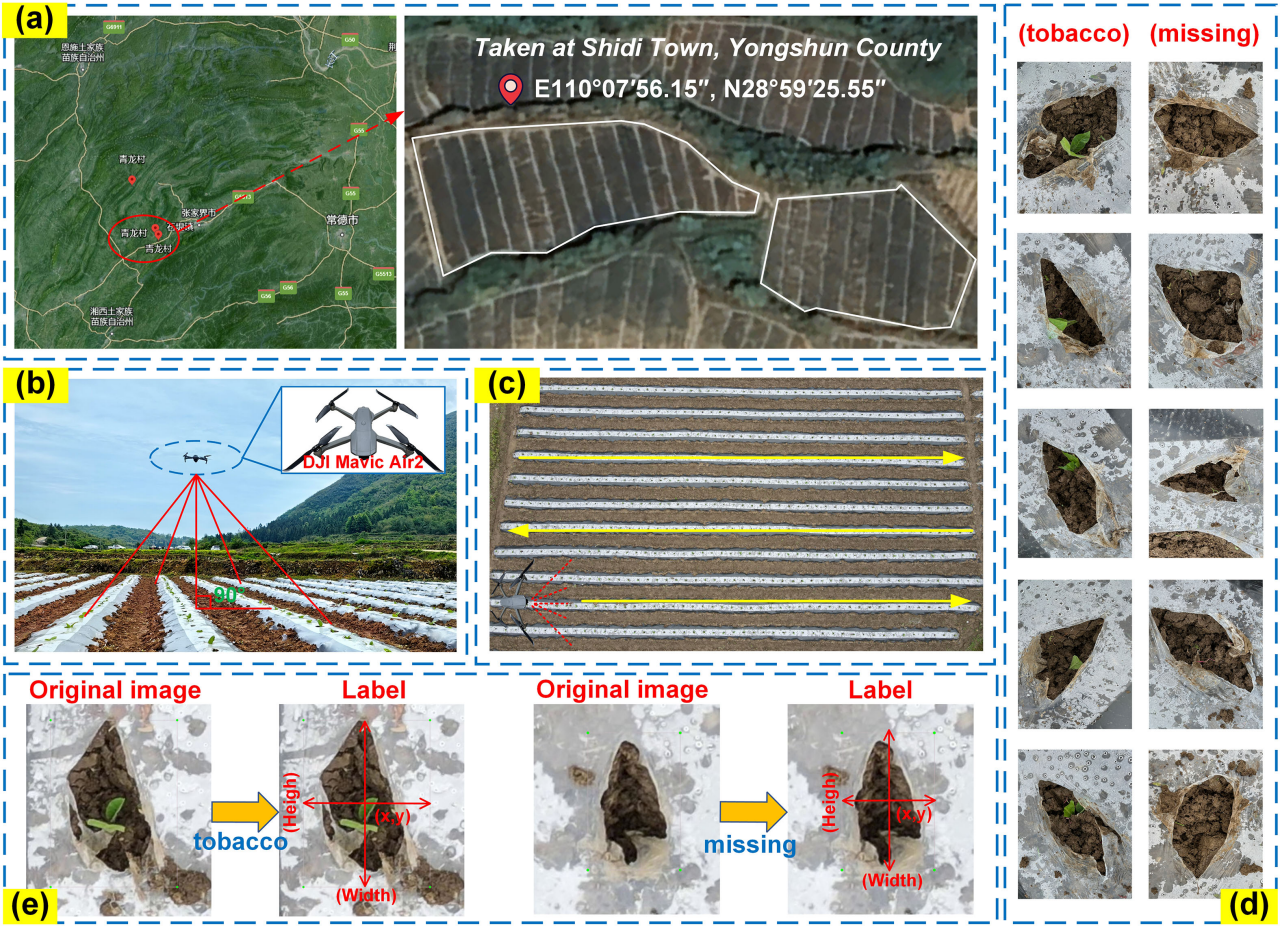
Detailed information on image dataset acquisition. **(a)** Data collection location; **(b)** Data acquisition site; **(c)** UAV flight trajectory; **(d)** Data set detection target indication; **(e)** Label production process.

The data collection site is located in Qinglong Village, Shidi Town, Yongshun County, Xiangxi Tujia and Miao Autonomous Prefecture, Hunan Province, within a humid, mid-subtropical mountainous climate zone. The site’s coordinates are 110°07′56.15″E and 28°59′25.55″N, as shown in **Figure 2a**. The cultivated tobacco variety in this area is “Yunyan 87.” The equipment and methods used for collecting tobacco seedling images are illustrated in **Figure 2b**. The drone model used for image and video data collection was a DJI Mavic Air 2, equipped with a DJI FC3170 camera. Data collection took place on April 23, 2024, at 9:00 AM. The drone ‘s flight trajectory is shown in **Figure 2c**. During image collection, the drone flew at a speed of 1.5m/s at an altitude of 10–15 meters, manually hovering at fixed points to ensure the overlapping area between images was less than 10%. For video data collection, the drone flew at a speed of 1.0m/s while maintaining the same altitude. After collection, the OpenCV library was used to extract one frame every 50 frames from selected videos, resulting in a total of 1,117 images and 20 transplanting video streams.

Due to factors such as gimbal camera settings and lighting conditions, the collected image data exhibited issues including underexposure, edge distortion, and low clarity. Manual cropping was performed to remove distorted edge regions, and images that did not meet the training requirements were discarded (Huang et al., 2024). The final dataset includes 1,054 images of tobacco seedlings for training the object detection model and 20 video streams for validating the proposed missed planting detection method. Examples of seedling-planted and missed-planting holes are shown in **Figure 2d**. Using the LabelImg tool (Wu et al., 2023), the images were annotated with two categories: “tobacco” for seedling-planted holes and “missing” for missed-planting holes, as shown in **Figure 2e**. The dataset contains 1,054 annotated images, with 17,698 “tobacco” bounding boxes and 2,609 “missing” bounding boxes.

### 2.3 Detection method based on improved YOLOv5s model

Before tracking and counting the number of seedling-planted and missed-planting holes, it is essential to accurately distinguish and identify these two target categories. YOLOv5 is a widely recognized object detection model, known for its stability and rapid detection capabilities, with applications in various agricultural fields (Ma et al., 2023; Zhang et al., 2024). The YOLOv5s version offers fast detection speed, high accuracy, and low memory usage, making it well-suited for deployment on mobile devices (Guo et al., 2024). Therefore, this study selects the YOLOv5s model as the base framework.

**Figure 3:**
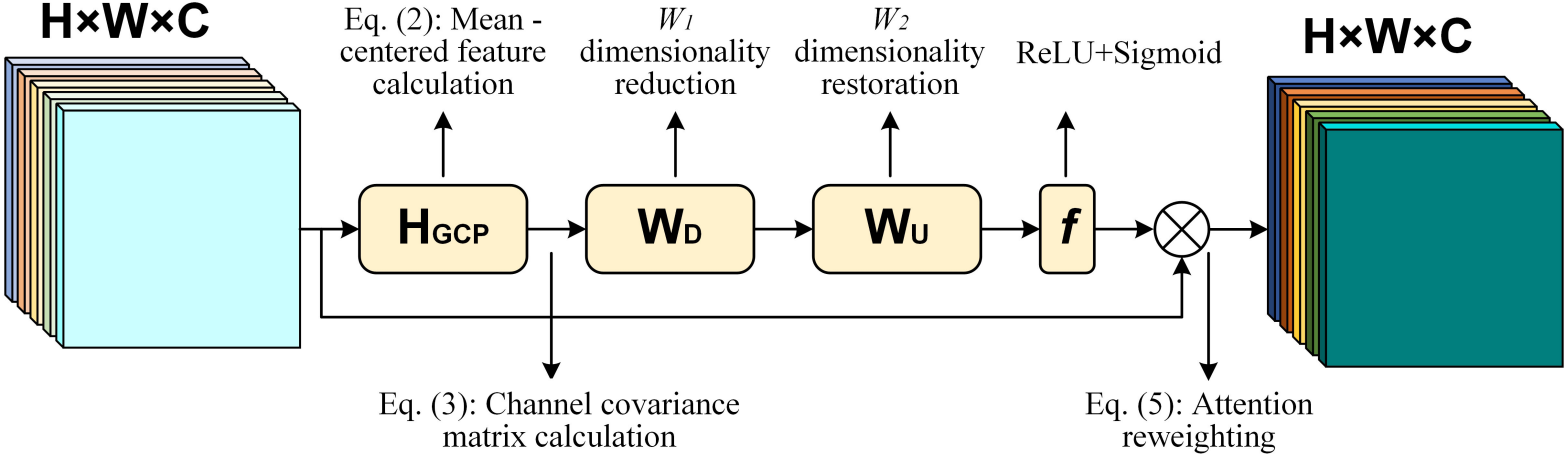
Structure of the SOCA module.

To enhance the feature extraction capability of the original YOLOv5s model for targets such as seedling-planted holes and missed-planting holes, the SOCA attention mechanism is introduced into the model. As shown in **Figure 3**, SOCA (Chen et al., 2020) employs covariance normalization to capture the correlation between channels. By adaptively realigning channel direction features using second-order feature statistics, SOCA improves the mapping of feature connections, enhances the model’s attention to relevant areas, and provides more discriminative representations. This ultimately boosts the identification performance of the YOLOv5s model.

The core idea of the SOCA module is to capture the relationships between channels by computing a covariance matrix. Specifically, given an input feature map $X\in R^{C\times H\times W}$ , where *C*, *H*, *W* represent the number of channels, height, and width, respectively, the first step is to calculate the mean-centered feature map $\hat{X}$ for each channel by subtracting the mean of that channel. The calculation method is shown in Equation (2)


(2) $${\hat{X}}_{i}=X_{i}-\frac{1}{HW}\sum_{h=1}^{H}\sum_{w=1}^{W}X_{i}(h,w)$$

Then, the covariance matrix $S\in R^{C\times C}$ is computed. The calculation method is shown in Equation (3)


(3) $$S_{ij}=\frac{1}{HW}\sum_{h=1}^{H}\sum_{w=1}^{W}{\hat{X}}_{i}(h,w){\hat{X}}_{j}(h,w)$$

Next, feature normalization and attention weight calculation are performed. The calculation method is shown in Equation (4)


(4) $$Z=\sigma (W_{2}\text{ReLU(}S)W_{1})$$

Finally, the attention weights *Z* are used to reweight the input feature map. The calculation method is shown in Equation (5)


(5) $$X^{\prime }=Z\cdot X$$

In this context, $X\in R^{C\times H\times W}$ represents the input feature map; $\hat{X}$ is the mean-centered feature map; $S\in R^{C\times C}$ is the covariance matrix between channels; ReLU(·) denotes the Rectified Linear Unit activation function; *W*_1_ and *W*_2_ are learnable weight matrices; σ(·) refers to the sigmoid activation function; Z is the attention weight matrix; and $X^{\prime }$ is the reweighted input feature map after applying the attention weights.

In this way, the SOCA module enhances the model’s ability to capture features, especially for small objects and complex backgrounds, thus improving detection performance.

**Figure 4:**
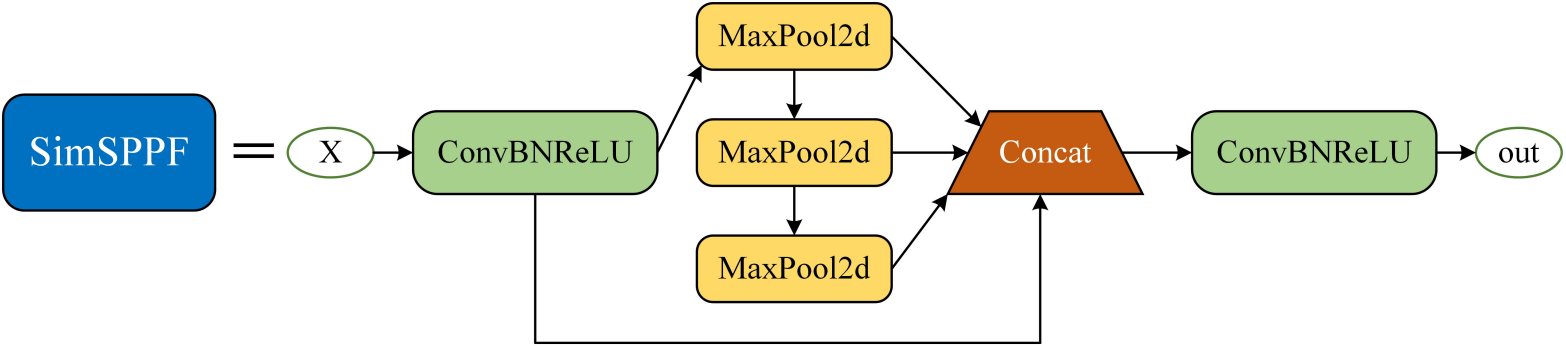
Structure of the SimSPPF module.

The Spatial Pyramid Pooling Fast (SPPF) in YOLOv5s uses fixed layers and scales, which causes the loss of small target details in the feature pyramid, adversely affecting small target perception and leading to inaccurate localization and missed detections. The Simplified Spatial Pyramid Pooling-Fast (SimSPPF) (Ye et al., 2024), an efficient and low-cost improvement, performs multi-scale feature extraction to effectively prevent the loss of local target information, thereby enhancing both feature extraction efficiency and detection accuracy. The structure of SimSPPF is shown in **Figure 4**. The SimSPPF module processes input feature *X* through a sequential yet branched workflow. Initially, *X* enters the ConvBNReLU module, where convolution, batch normalization, and ReLU activation are integrated to extract preliminary features. Post-ConvBNReLU, the feature stream splits: one branch directly proceeds, while the other undergoes three successive MaxPool2d operations. Each MaxPool2d step downsamples features spatially, enabling capture of information at diverse scales. These multi - scale features—comprising the direct branch and the three pooled branches—then converge at the Concat module, which fuses them via channel - wise concatenation. Finally, the fused features pass through another ConvBNReLU for further integration and nonlinear transformation, yielding the output “out”. Compared to traditional SPPF, SimSPPF significantly improves localization accuracy and detection reliability of small targets.

**Figure 5:**
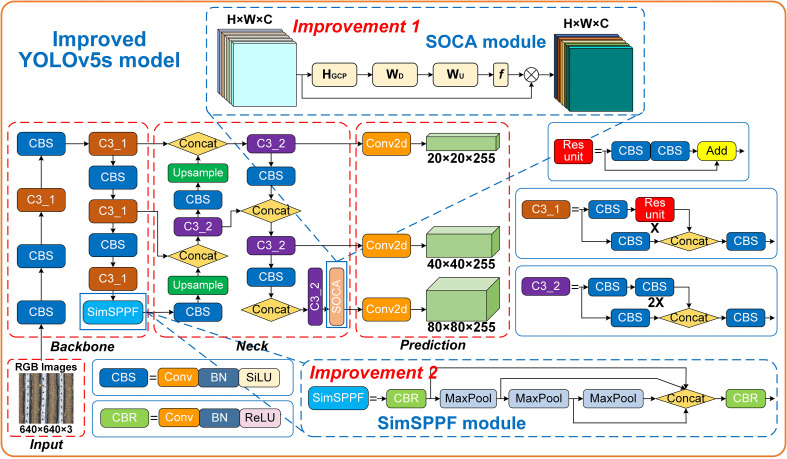
Improved YOLOv5s model network architecture.

In summary, the improved YOLOv5s model enhances small target detection accuracy by introducing the SOCA attention mechanism and SimSPPF. The SOCA embedded in the final layer of the feature extraction network, improves the recognition of key features for seedling-planted and missed-planting holes, thereby reducing false positives and false negatives in complex environments. SimSPPF replaces the traditional SPPF, utilizing multi-scale feature extraction to prevent information loss, enhancing both detection efficiency and accuracy. The improved YOLOv5s significantly improves target localization and detection reliability, even in the presence of complex background interference. The architecture of the improved YOLOv5s model is shown in **Figure 5**.

### 2.4 Tracking of seedling-planted and missed-planting holes based on DeepSORT algorithm

**Figure 6:**
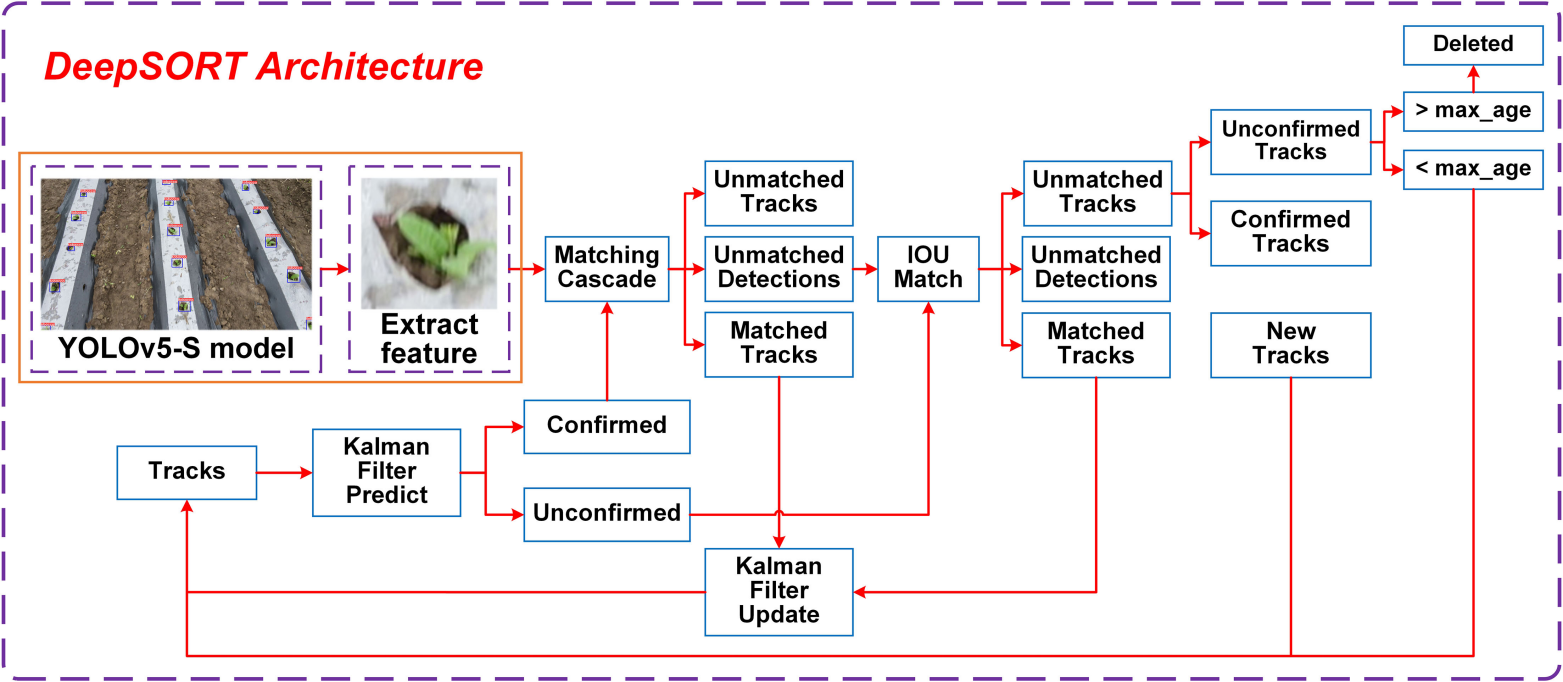
Dynamic tracking architecture of the DeepSORT algorithm.

To prevent duplicate counting issues during the dynamic tracking of seedling-planted and missed-planting holes and to improve counting accuracy, Multi-Object Tracking (MOT) (Wu et al., 2023) technology is employed following target detection, utilizing the DeepSORT algorithm. The primary task of this algorithm is to detect objects in consecutive video frames and assign a unique identifier to each object, ensuring consistency across the video sequence. Building upon the SORT algorithm, DeepSORT incorporates a Re-Identification (Re-ID) model to address target identifier recognition challenges. The Re-ID model determines the target’s unique identifier by calculating its similarity across multiple frames and applies a cascade matching technique to enhance feature matching, thereby improving the robustness of DeepSORT’s object tracking. The processing flow of the DeepSORT algorithm is shown in **Figure 6**.

The DeepSORT tracking process matches detection boxes from the current frame with trackers from the previous frame based on the prediction results from the Kalman filter. First, cascade feature matching is performed between the detection boxes and confirmed state trackers. Next, for the detection boxes, trackers, and unconfirmed state trackers that were not successfully matched during the cascade step, Intersection over Union (IoU) matching is applied. Finally, the successfully matched trajectories are updated using the Kalman filter, completing the tracking process (Huang et al., 2023).

### 2.5 Statistics of seedling-planted and missed-planting holes based on cross-line counting.

**Figure 7:**
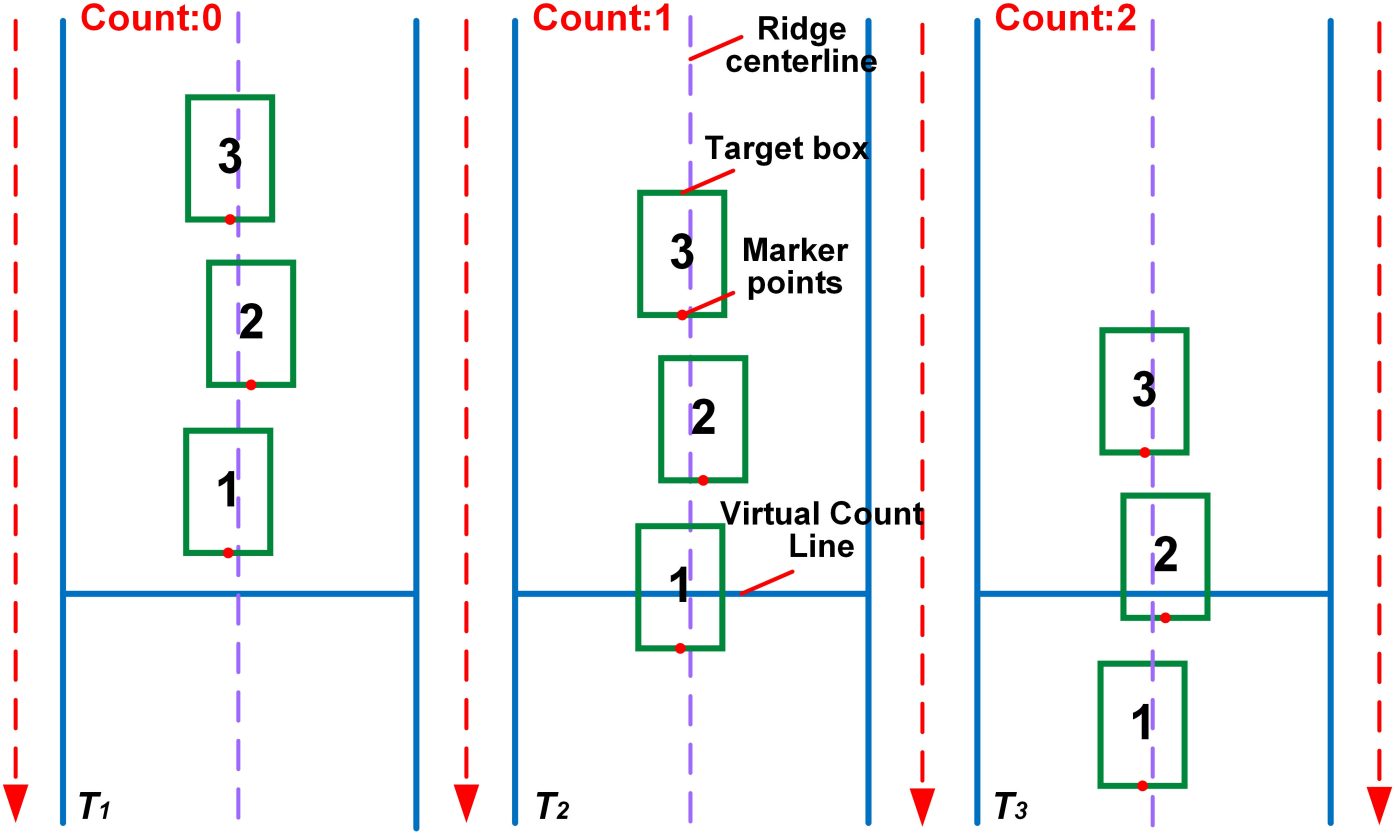
Schematic diagram of line crossing counting principle.

To achieve the classified counting of seedling-planted holes (T1) and missed-planting holes (T2), a line-crossing counting method is embedded based on the DeepSORT algorithm, which pre-classifies the two types of targets by assigning unique tracking IDs. The virtual counting line is set at the optimal angle and distance. When a target completely appears within the bounding box, a marker point is placed at the midpoint of its lower bounding box (red for T1 and blue for T2). When a marker point crosses the counting line for the first time, the quantity of the corresponding category is incremented respectively (T1 triggers the count of seedling-planted holes to increase by 1, and T2 triggers the count of missed-planting holes to increase by 1). Moreover, the tracking ID is used to avoid repeated counting of the same target. Finally, the cumulative quantities of the two types of targets are presented in a visual form. **Figure 7** illustrates the counting principle for one of the target categories.

### 2.6 Model training and evaluation

#### 2.6.1 Training environment and parameters

Model training and testing were conducted on a Windows 11 system. The hardware configuration included an Intel^®^ Core™ i9-13900K CPU running at 4.90 GHz, 32 GB of 3200 MHz memory, and an NVIDIA GeForce RTX 4080 GPU with 16 GB of VRAM. The official open-source YOLOv5s code from Ultralytics was adopted as the baseline for engineering improvements.

The experiment utilized Stochastic Gradient Descent (SGD) as the optimizer to improve the neural network’s performance and accelerate the fitting process. Optimal hyperparameters were determined using the grid search algorithm, including an initial learning rate of 0.01 and a weight decay coefficient of 0.0005. The training strategy incorporated a momentum-based gradient descent algorithm with a momentum value of 0.937, and the number of epochs was set to 300. Input images were uniformly resized to 640×640 pixels, and the batch size was set to 32. A cosine annealing algorithm was used to adjust the learning rate dynamically. The pre-trained YOLOv5s.pt file, trained on the large-scale COCO2017 dataset, was used as initialization weights.

#### 2.6.2 Evaluation metrics

This paper evaluates the model from two perspectives: model complexity and recognition performance. The performance metrics include Precision, Recall, mean average precision (mAP), inference time, and model size. Recall refers to the proportion of correctly identified positive samples in all actual positive samples. mAP synthesizes the average precision (AP) of each category. AP reflects the balance between Precision and Recall under different IoU thresholds. The higher the value, the better the performance of the model. Among these metrics, Precision, Recall, and mAP serve as comprehensive indicators of the model’s recognition performance, with higher values denoting better performance. Inference time and model size evaluate the model’s efficiency, with lower values indicating greater efficiency. As this study focuses on the model’s ability to distinguish and recognize targets as well as its complexity, other evaluation metrics of the YOLOv5s model are not considered. The calculation of Precision, Recall, AP and mAP refers to Equations (6–9).


(6) $$P=\frac{TP}{TP+FP}\times 100\%$$

(7) $$R=\frac{TP}{TP+FN}\times 100\%$$

(8) $$AP=\int_{0}^{1}P(R)dR$$

(9) $$mAP=\frac{\sum_{i=1}^{n}AP_{i}}{n}\times 100\%$$

In the formula, *TP* represents the number of correctly detected objects, *FP* represents the number of incorrectly detected objects, *FN* represents the number of objects missed by the algorithm, and *n* represents the number of detection categories. In this study, n = 2.

The performance of the tracking method is evaluated using the metrics missed detection Rate *M_m_*, false detection rate *M_f_*, multi-object tracking Accuracy (MOTA), and Frames Per Second (FPS). A higher MOTA value signifies better tracking performance. FPS is a key indicator of the model’s processing speed and real-time performance, with higher values reflecting faster data processing. Conversely, lower *M_m_* and *M_f_* values reflect improved tracking performance. The calculation of these metrics refers to Equations (10–12).


(10) $$M_{m}=\frac{FN}{FN+TP}\times 100\%$$

(11) $$M_{f}=\frac{FP}{FP+TN}\times 100\%$$

(12) $$MOTA=1-\frac{\sum_{t}NM_{m}+NM_{f}+IDS}{\sum_{t}GT_{t}}$$

In the formula, *TN* represents the number of correctly detected objects of the other class; *NM_m_* denotes the number of missed detections at time *t*, *NM_f_* represents the number of false detections at time *t*, *IDS* indicates the number of ID switches, and *GTt* represents the number of ground truth objects at time *t*.

In the field operation experiment for detecting and evaluating the missed transplanting rate, the detection accuracy, denoted as *D_t_*, is used as the evaluation metric. The specific calculation method refers to Equation (13).


(13) $$D_{t}=\frac{R_{\text{model}}}{R_{\text{truth}}}\times 100\%$$

Where *R*_model_ represents the missed transplanting rate value of the seedling transplanter based on the model’s counting results, and *R*_truth_ represents the missed transplanting rate value of the seedling transplanter based on the manual counting results.

## 3 Results and discussions

### 3.1 Model evaluation test

#### 3.1.1 Comparison of YOLO series models

To select a better base model, the performance of various YOLO models was evaluated on the same self-constructed dataset (as described in Section 2.2), with each model trained for 120 epochs, as shown in **Table 1**. The YOLOv5s model outperformed other mainstream models in the YOLO series across precision, recall, mean average precision (mAP), model size, and inference time, achieving 75.1%, 75.4%, 75.8%, 14.1MB, and 0.0168s, respectively. Its performance is comparable to that of the YOLOv11s model. However, considering the need for lightweight deployment on edge devices in subsequent models, YOLOv5s was ultimately chosen as the base model.

**Table 1:** Performance comparison of YOLO series models in the same dataset.

Model	Precision/%	Recall/%	mAP/%	Model size/MB	Running time/s
YOLOv3-tiny	71.2	70.5	71.5	58.2	0.0579
YOLOv4-tiny	72.2	72.1	72.8	22.6	0.0297
YOLOv5s	75.1	75.4	75.8	14.1	0.0168
YOLOv7-X	71.4	72.1	71.5	72.0	0.0612
YOLOv8s	72.6	72.2	72.9	42.7	0.0510
YOLOv11s	74.9	75.1	75.2	19.6	0.0178

**Figure 8:**
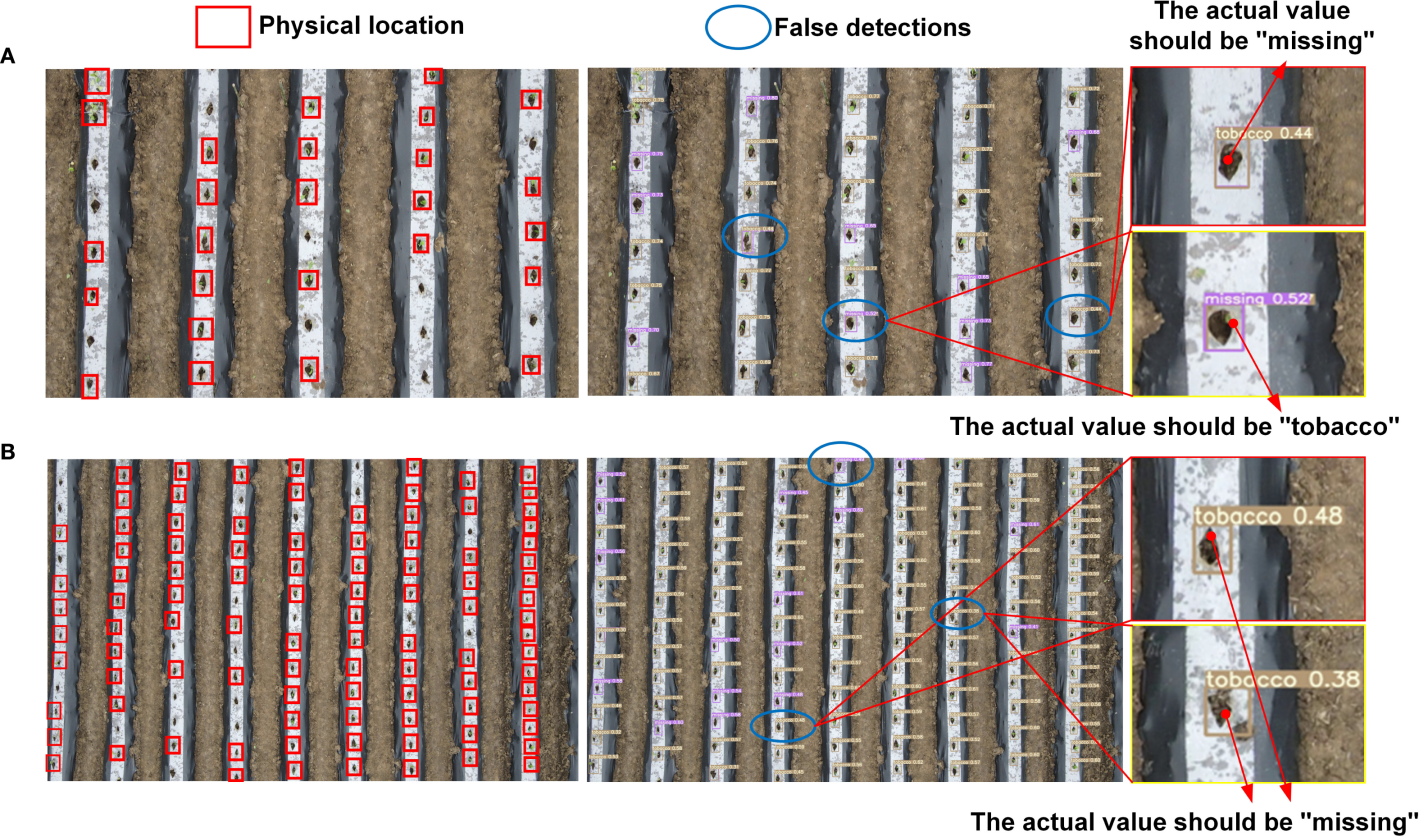
Performance of the original YOLOv5s in more complex scenarios. **(A)** Visualization of conventional scene error detection and missed detection; **(B)** More complex scene misdetection and missed detection visualization.

The test results from the test set are illustrated with two selected images. In the first column, a red box is used to mark the actual seedling-planted holes, with the remaining unmarked areas representing missed-planting holes. As shown in **Figure 8**, the YOLOv5s model’s detection results closely match the ground truth annotations, effectively distinguishing between seedling-planted holes and missed-planting holes. However, in certain challenging scenarios with complex backgrounds or very small seedlings, the model misclassified the two states. The overall Precision was 75.1%, with a Recall of 75.4%, indicating potential for further improvement.

#### 3.1.2 Model ablation experiments and analysis

To enhance the model’s ability to focus on features associated with seedling-planted and missed-planting holes, the SOCA attention mechanism was integrated into the feature network, improving recognition accuracy in complex scenarios. Additionally, to strengthen the model’s ability to capture color features, the SimSPPF module was incorporated to expand the feature extraction network, replacing the original SPPF. Ablation experiments were conducted to assess each improvement strategy individually, and the results are shown in **Table 2**.

**Table 2:** Results of ablation experiments based on different improvement strategies.

Model	Precision/%	Recall/%	mAP/%	Model size/MB	Running time/s
YOLOv5s	75.1	75.4	75.8	14.1	0.0168
YOLOv5s+SOCA	77.2	77.8	77.4	14.2	0.0172
YOLOv5s+SimSPPF	76.5	76.6	76.2	14.1	0.0169
YOLOv5s+SOCA+ SimSPPF (Ours)	79.0	79.6	81.1	14.2	0.0172

The results of the ablation experiment demonstrate that integrating the SOCA attention mechanism and replacing the SPPF module with SimSPPF in YOLOv5s yielded the most significant improvements in detection performance. Precision and mAP reached 79.0% and 81.1%, respectively representing increases of 3.9% and 5.3% compared to the original YOLOv5s. These improvements highlight that the enhanced accuracy of the modified YOLOv5s model is due to the combined effects of the SOCA attention mechanism and the SimSPPF module, which together enhance the model’s ability to discriminate target features and improve recognition in UAV imagery. Despite a slight increase in model size (0.1 MB), the runtime remained at 0.0172 seconds, meeting the performance requirements for agricultural applications.

**Figure 9:**
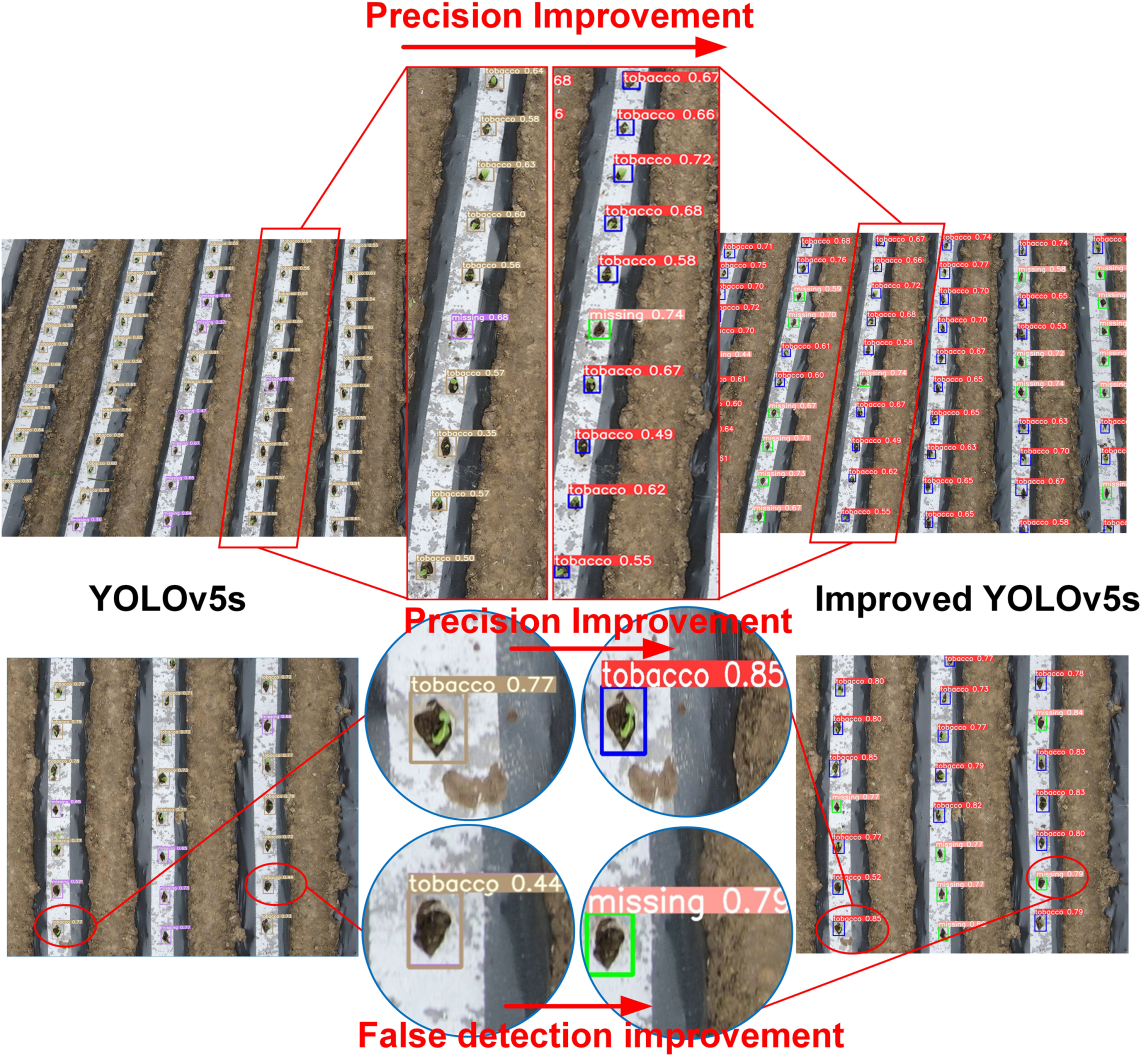
Performance improvement details of YOLOv5s before and after enhancement in some complex scenarios.

**Figure 9** illustrates the improvements in detection performance before and after modifications to the YOLOv5s model. As shown in **Figure 9**, the improved YOLOv5s model outperforms the original model in randomly selected scenes, exhibiting a notable increase in precision. Furthermore, the improved model excels in minimizing false detections, effectively distinguishing between seedling-planted holes and missed-planting holes, even in challenging scenarios.

#### 3.1.3 Performance evaluation of the improved YOLOv5s model

To further evaluate the recognition capability of the improved YOLOv5s model, frames were extracted from the video stream at intervals of 50 frames using the OpenCV library, resulting in a test set of 50 static images. The evaluation results are presented in **Table 3**. The test set included a total of 2,140 seedling-planted holes and 602 missed-planting holes. The proposed model successfully detected 1,996 seedling-planted holes and 563 missed-planting holes. Precision and Recall for seedling-planted holes were 92.9% and 85.9%, respectively, while for missed-planting holes, they were 91.4% and 84.6%. These results further demonstrate the model’s robust generalization ability and reliability.

**Table 3:** Evaluation results of the improved YOLOv5s on the test set.

Class	Ground truth	Proposed method	TP	FP	FN	Precision/%	Recall/%
tobacco	2140	1996	1839	141	301	92.9	85.9
missing	602	563	509	48	93	91.4	84.6

**Figure 10:**
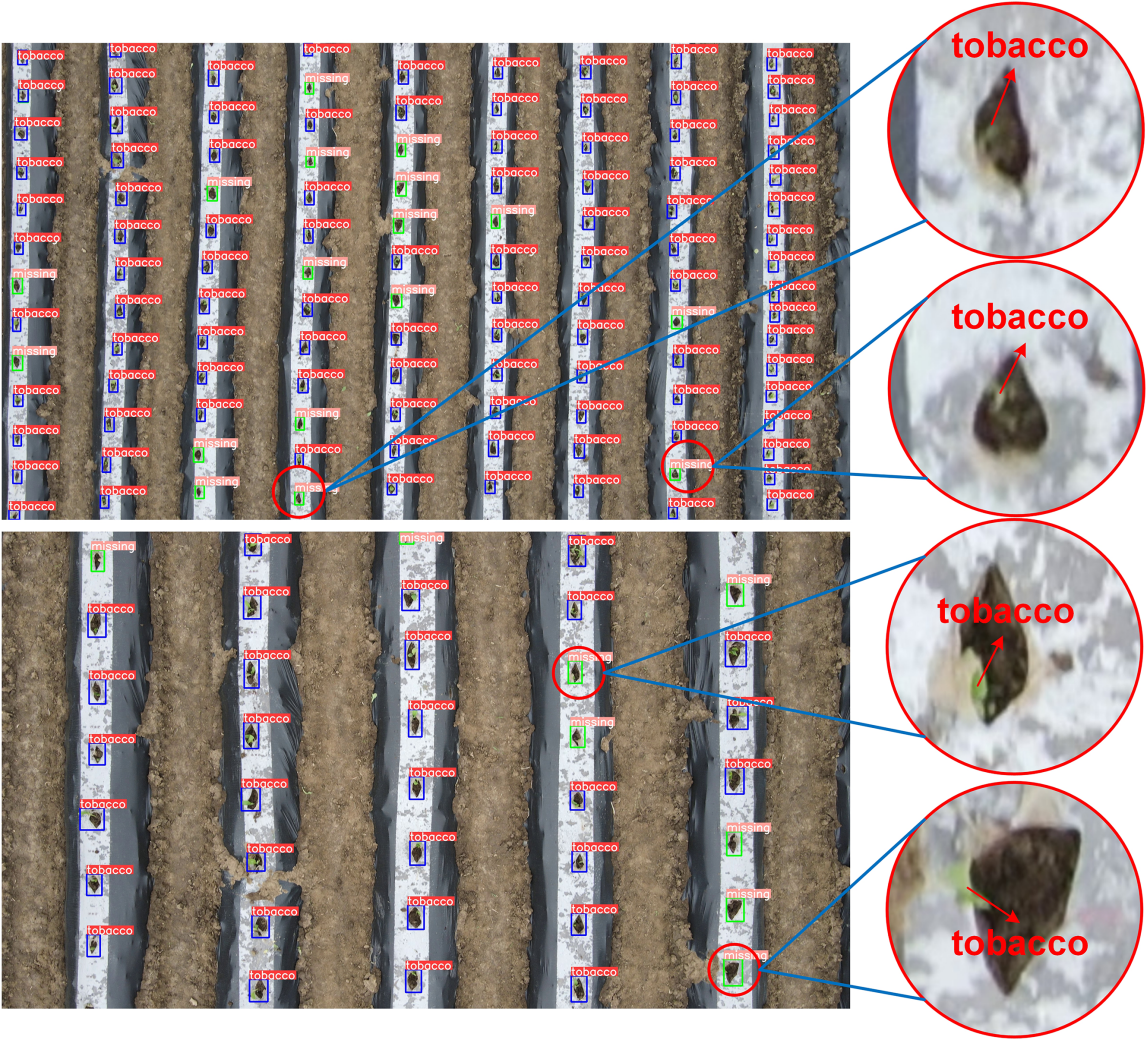
Examples scenarios of misdiagnosis and omission.

Despite improvements, false detections still occur, as shown in **Figure 10**. This is primarily due to two factors: first, extreme lighting conditions and reflections from the film surface in high-altitude images contribute to false positives; second, the variation in seedling quality, with some weak seedlings being transplanted. In high-altitude UAV imagery, the pixel points of the seedlings are extremely limited, and few features can be extracted, making it difficult to distinguish between seedling-planted holes and missed-planting holes, thus leading to false detections.

### 3.2 Comparison of tracking algorithm performance

To evaluate the performance of the improved YOLOv5s model combined with the DeepSORT tracking algorithm for tracking and counting transplanted tobacco seedlings, a comparative experiment was conducted. The experiment compared the counting results of the original YOLOv5s model with those of the enhanced version, which included DeepSORT, using UAV-captured videos of transplanted tobacco seedlings. The results of this experiment are presented in **Table 4**.

**Table 4:** Performance comparison of DeepSORT combined with YOLOv5 before and after improvement.

Models	*M_m_*/%	*M_f_*/%	MOTA/%	FPS/(f·s^−1^)
YOLOv5s+DeepSORT	6.6%	15.2%	74.1%	15.9
Improved YOLOv5s+DeepSORT	4.1%	9.1%	78.5%	19.9

**Figure 11:**
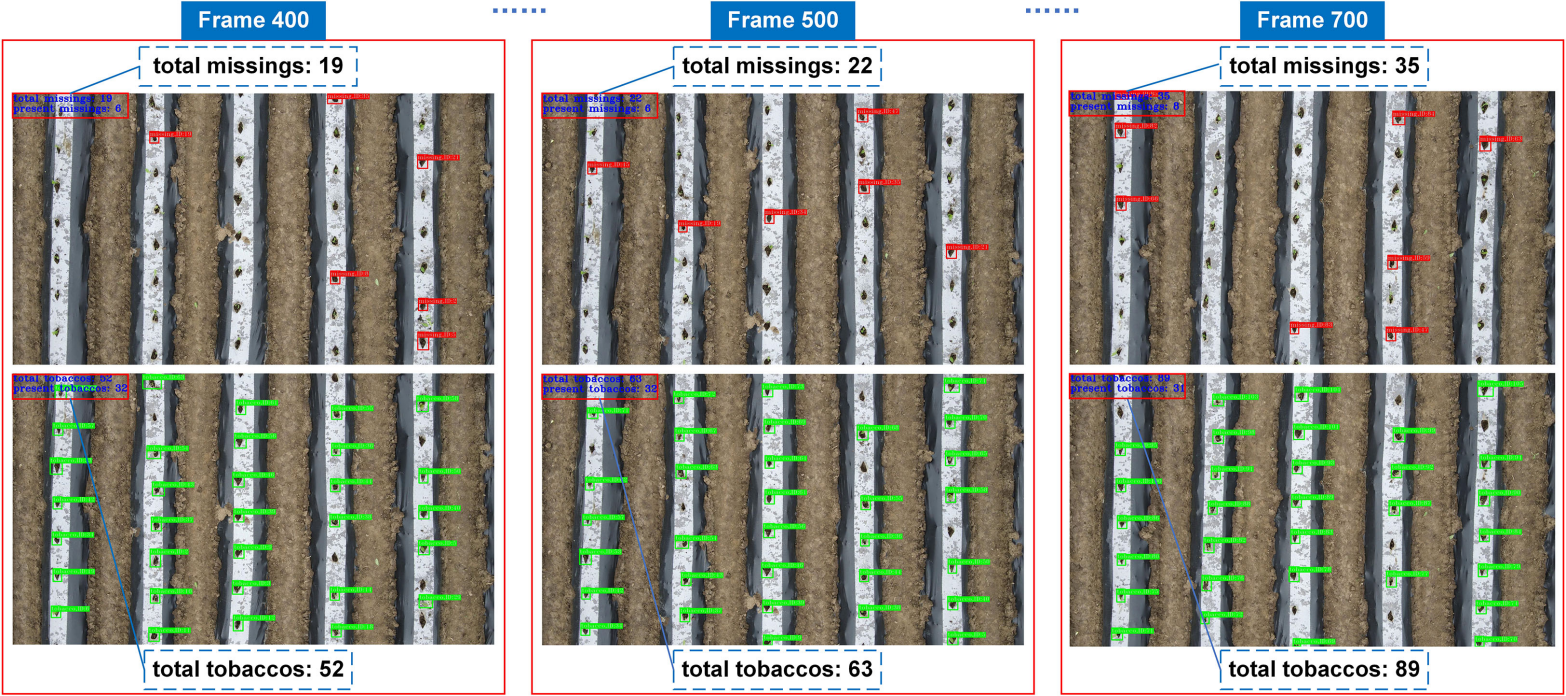
Visualization results of the tracking and counting process.

As shown in **Table 4**, integrating the improved YOLOv5s model with the DeepSORT tracking algorithm significantly enhances tracking and counting performance. The missed detection rate *M_m_* and false detection rates *M_f_* during target tracking were 4.1% and 9.1%, respectively, reflecting reductions of 2.5% and 6.1% compared to the original YOLOv5s, with the FPS of 19.9. However, the MOTA evaluation results indicate that neither DeepSORT nor other advanced tracking algorithms can fully resolve the issue of identifier switching. Due to the small size of tobacco seedling targets, distinguishing between seedling-planted and missed-planting holes remains challenging, leading to multiple identifiers being assigned to the same target across frames, which results in duplicate counting. Therefore, using either the maximum identifier count or the total number of identifiers as the final count is inaccurate. To improve accuracy, this study proposes a method for automatically counting seedling-planted and missed-planting holes. In this method, the count is incremented by one each time the lower edge of the detection box for a seedling-planted or missed-planting hole crosses a virtual counting line. The cumulative totals for both types of holes are displayed in real-time on the video, as shown in **Figure 11**.

From **Figure 11**., it can be seen that the tracking and counting of the seedling-planted and missed-planting holes respectively, the detection of the two types of targets in the same frame does not appear wrong detection and missed detection, and the target counting results shown in the upper left corner are consistent with the actual results.

### 3.3 Detection and counting system platform

**Figure 12:**
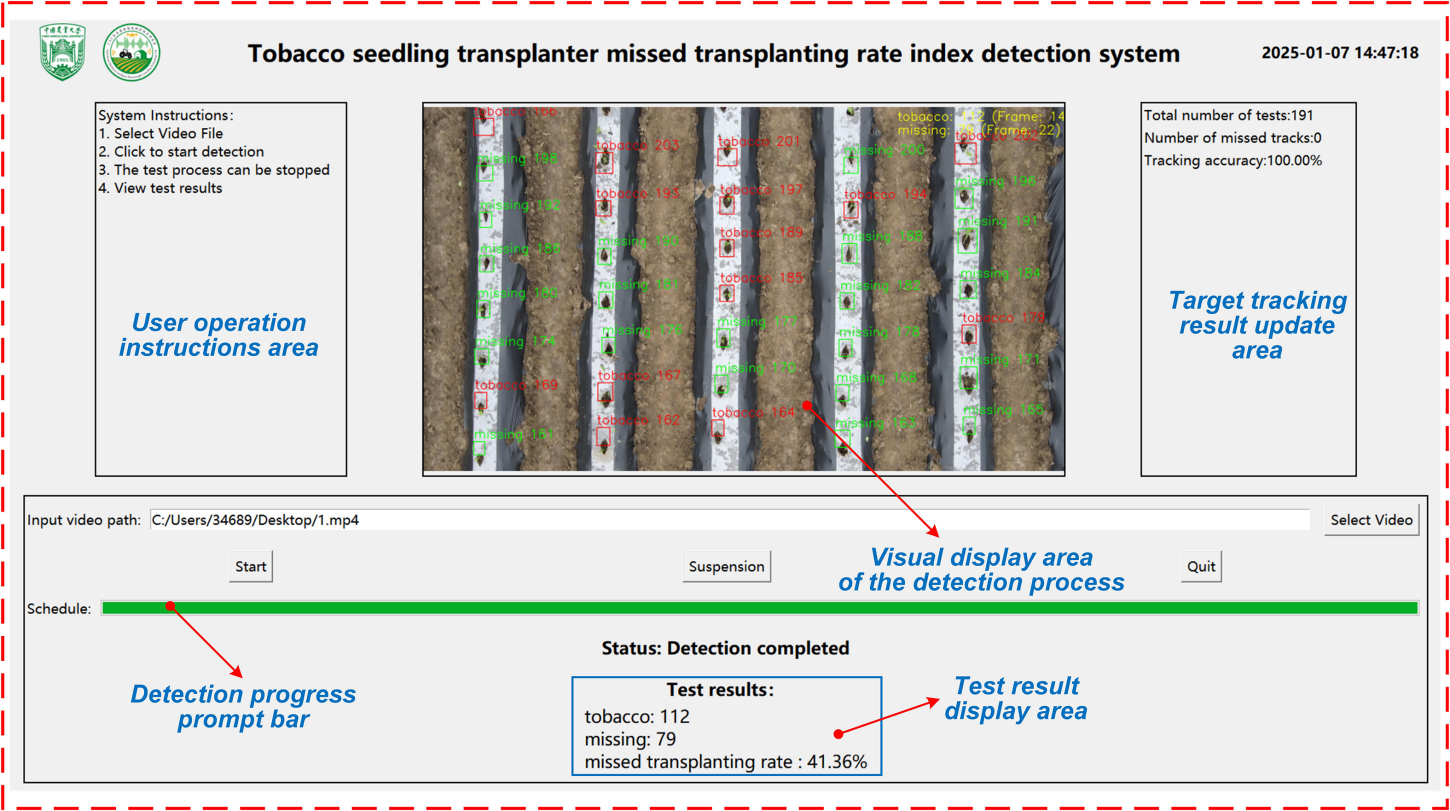
Performance index detection system for missed transplanting rate.

To efficiently and accurately assess the number of seedling-planted and missed-planting holes in tobacco mechanized transplanting operations, this study implements an improved YOLOv5s model, DeepSORT, and crossing-line counting algorithms on a PC platform. A performance metric detection system for the missed transplanting rate of tobacco seedling transplanters is developed based on the PyQT5 framework, with the PC user interface designed using QT tools, as shown in **Figure 12**. Upon initiating the detection process, users can upload a video via the interface. The system automatically loads the video and applies the optimized deep learning model for analysis. Trained on large-scale data, the model efficiently and accurately identifies the number of seedling-planted and missed-planting holes, enabling the calculation of the missed transplanting rate. During detection, the system dynamically displays visualized results of the video processing. Once detection is complete, the interface presents statistical data on seedling-planted and missed-planting holes, along with the missed transplanting rate, offering an intuitive representation of the seedling transplanter’s performance. Furthermore, the generated video and related data are automatically stored in the backend database for future querying, statistics, and analysis.

### 3.4 Qualitative analysis of missed transplanting rate detection

**Figure 13:**
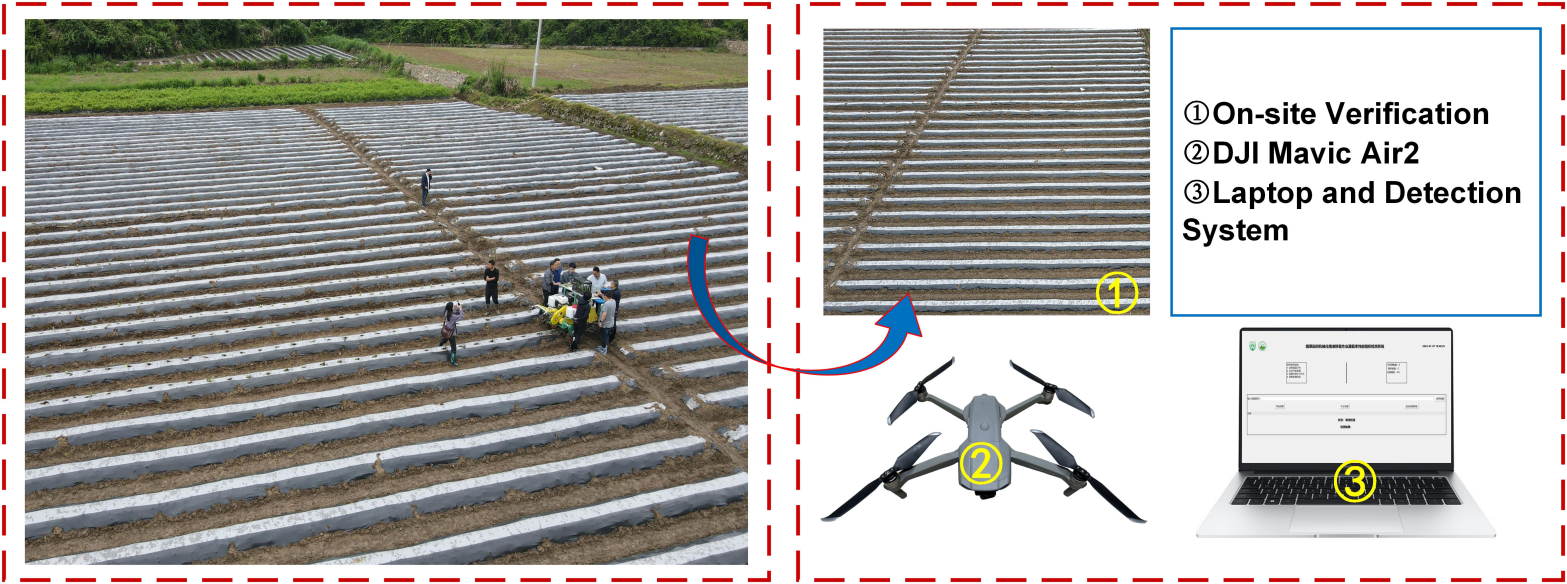
Field test site and the equipment used.

**Figure 14:**
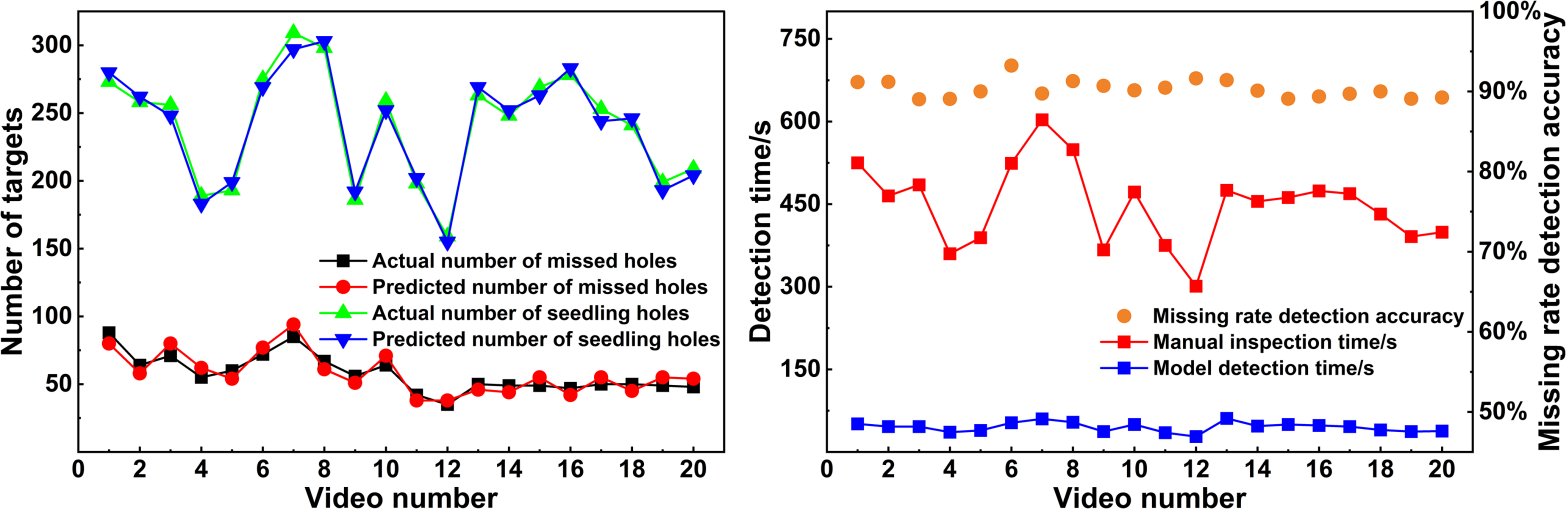
Video detection results comparison.

To validate the feasibility of the counting method, a mechanized tobacco transplanting operation test was conducted in June 2024 in Yongshun County, Xiangxi Tujia and Miao Autonomous Prefecture, Hunan Province, using a self-propelled seedling transplanter. The test site and the equipment used are shown in **Figure 13**. Following the transplanting, 20 dynamic video segments captured by drones were evaluated. First, three skilled operators independently counted the number of the objects in the 20 video segments. The average value of the counts obtained by the three operators was taken as the actual number of seedling-planted and missed-planting holes in the aerial videos. During counting, the operators played the field videos frame by frame, recording the number of seedling-planted and missed-planting holes in the first frame along with the start time. Subsequently, they recorded the number of newly appearing seedling-planted and missed-planting holes in each subsequent frame until the end of the video. This process yielded the total number of seedling-planted and missed-planting holes in the video, as well as the time taken for manual counting. Next, the proposed model was used to determine the number of seedling-planted and missed-planting holes in the videos and the time consumed by the algorithm for counting. The results were compared with the manual counts to test the detection accuracy and efficiency of the missed transplanting rate. The results are shown in **Figure 14**.

As shown in **Figure 14**, using the algorithmic model to analyze the 20 videos collected from the test area, the average detection accuracy *D_t_* of the missed transplanting rate was 90.28%, and the detection efficiency of the model was 10 times that of manual counting. These results demonstrate that the proposed method for detecting the missed transplanting rate significantly improves the efficiency of evaluating the missed transplanting rate following the operation of the tobacco seedling transplanter.

## 4 Discussions

The method proposed in this paper still faces several issues that deserve discussion in practical applications. First, the flight altitude and shooting angle of the drone during data collection can affect the application performance of the method. To balance the efficiency and accuracy of missed transplanting detection, the drone’s camera should be perpendicular to the ground, with a shooting angle of -90°, and a flight altitude of 15–20 meters. The optimal field of view occurs when 6–8 rows of tobacco seedlings are visible in the drone’s video imagery, which ensures high applicability of the proposed method. Secondly, under specific agronomic requirements such as film-covered well-shaped transplanting and actual background interference, while focusing on the recognition accuracy during target identification, attention should also be given to reducing false positives. The improved YOLOv5s model proposed in this paper effectively reduces the probability of false positives and can better distinguish between seedling-planted holes and missed-planting holes. It also shows significant improvements in both Precision and mAP. Additionally, for large-scale transplanting operations by tobacco seedling transplanter, where missed planting detection is required, the detection efficiency of the proposed method is 10 times that of manual counting, meeting the requirement for rapid detection and practical application. However, the method still has certain limitations. So far, the training set used represents the field conditions of tobacco transplanting with film-covered soil in specific regions, and the dataset covers a relatively limited range of scenarios. Therefore, the performance of the trained model may decrease when applied to transplanting scenes outside of these conditions. Adapting the model to meet the needs of different tobacco transplanting scenarios will be a key focus of our future research. Meanwhile, this study uses drones as data collection devices, and the proposed method is not well-suited for areas with restrictions on drone flights. Furthermore, to improve the real-time nature and convenience of the detection process, the algorithm could be deployed on the drone’s edge platform, and a smartphone interface could be developed for real-time image transmission and display, enabling real-time detection and providing system-level technical support for tobacco seedling transplanter operation performance monitoring.

## 5 Conclusions

This study proposes an intelligent evaluation method for the missed transplanting rate of tobacco seedling transplanters, based on drone video imagery and an algorithm framework incorporating the improved YOLOv5s, DeepSORT, and line-crossing counting methods. This approach enhances the YOLOv5s model by adding a SOCA attention mechanism and replacing the original SPPF structure with the SimSPPF structure, which improves the model’s feature extraction capability. The method also combines the DeepSORT algorithm and line-crossing counting principle to recognize and dynamically count seedling-planted and missed-planting holes, thereby calculating the missed transplanting rate. The results show that, in terms of detection performance, the improved YOLOv5s model outperforms the original YOLOv5 by 3.9% in Precision and 5.3% in mAP. In tracking accuracy, combining the improved YOLOv5s with DeepSORT reduces the missed detection rate *M_m_* and false detection rate *M_f_* by 2.5% and 6.1%, respectively, compared to the original YOLOv5s model. Testing the proposed method on an independent dataset, the detection Precision and Recall for seedling-planted holes (tobacco) were 92.9% and 85.9%, respectively, while for missed-planting holes (missing), the Precision and Recall were 91.4% and 84.6%, respectively. Furthermore, when the proposed method was applied to assess 20 drone dynamic video images captured in field experiments of tobacco seedling transplanters, the average missed transplanting rate detection accuracy *Dt* was 90.28%, and the detection efficiency was 10 times that of manual counting. Therefore, this method significantly improves the efficiency of missed transplanting rate detection for tobacco seedling transplanters, providing a technical reference for the evaluation of seedling transplanter performance in large-scale transplanting operations.

## Data Availability

The original contributions presented in the study are included in the article/supplementary material. Further inquiries can be directed to the corresponding author/s.

## References

[B1] Bao W. X. Xie W. J. Hu G. S. Yang X. J. Su B. B. (2023). Wheat ear counting method in UAV images based on TPH-YOLO. Trans. Chin. Soc. Agric. Eng. 39, 155–161. doi: 10.11975/j.issn.1002-6819.202210020

[B2] Barreto A. Lottes P. Ispizua Yamati F. R. Baumgarten S. Wolf N. A. Stachniss C.. (2021). Automatic UAV-based counting of seedlings in sugar-beet field and extension to maize and strawberry. Comput. Electron. Agric. 191, 106493. doi: 10.1016/j.compag.2021.106493

[B3] Bewley A. Ge Z. Ott L. Ramov F. Upcroft B. (2016). “Simple online and realtime tracking,” in 2016 IEEE International Conference on Image Processing (ICIP), Phoenix, AZ, USA. 3464–3468. doi: 10.1109/ICIP.2016.7533003

[B4] Chen Y. Chen Y. Xue J. Yang W. Liao Q. (2020). “Lightweight single image super-resolution through efficient second-order attention spindle network,” in 2020 IEEE International Conference on Multimedia and Expo (ICME), London, UK. 1–6. doi: 10.1109/ICME46284.2020.9102946

[B5] Cui J. Zheng H. Zeng Z. Yang Y. Ma R. Tian Y.. (2023). Real-time missing seedling counting in paddy fields based on lightweight network and tracking-by-detection algorithm. Comput. Electron. Agric. 212, 108045. doi: 10.1016/j.compag.2023.108045

[B6] Guo J. Wang R. Nan J. Li X. Jiao C. (2024). YOLOv5 model integrated with GRN attention mechanism for insect pest recognition. Trans. Chin. Soc. Agric. Eng. 40, 159–170. doi: 10.11975/j.issn.1002-6819.202310226

[B7] Huang C. Hua X. Huang S. Lu Z. Dong J. Zhang J.. (2023). Regenerated buds tracking and regenerative ability evaluation of ratooning rice using Micro-CT imaging and improved DeepSORT. Trans. Chin. Soc. Agric. Eng. 39, 165–174. doi: 10.11975/j.issn.1002-6819.202302033

[B8] Huang X. M. Zhang W. Qiu T. Zhu Y. Z. Xu S. X. Li W. C. (2024). Rapeseed seedling detection and counting based on UAV videos. Trans. Chin. Soc. Agric. Eng. 40, 147–156. doi: 10.11975/j.issn.1002-6819.202312224

[B9] Ji J. Chen K. Jin X. Wang Z. Dai B. Fan J.. (2020). High-efficiency modal analysis and deformation prediction of rice transplanter based on effective independent method. Comput. Electron. Agric. 168, 105126. doi: 10.1016/j.compag.2019.105126

[B10] Jiang Z. Zhang M. Wu J. Jiang L. Li Q. (2022). Real−time monitoring method for rape blanket seedling transplanting and omission based on video image SSplicing. J. Agric. Mechanization Res. 44, 189–195. doi: 10.13427/j.cnki.njyi.2022.09.021

[B11] Jin X. Zhao K. Ji J. Du X. Ma H. Qiu Z. (2018). Design and implementation of intelligent transplanting system based on photoelectric sensor and PLC. Future Generation Comput. Syst. 88, 127–139. doi: 10.1016/j.future.2018.05.034

[B12] Li J. P. Sun J. G. Sun G. W. Tan B. K. Chen G. Q. Chen Z. G.. (2023). Effects of well-cellar specifications and filling time on growth, yield, and quality of flue-Cured tobacco in well-cellar transplanting technology. J. Anhui Agric. Sci. 51, 31–32. doi: 10.3969/j.issn.0517-6611.2023.05.008

[B13] Li J. Zhu Z. Liu H. Su Y. Deng L. (2023c). Strawberry r-CNN: recognition and counting model of strawberry based on improved faster r-CNN. Ecol. Inform. 77, 102210. doi: 10.1016/j.ecoinf.2023.102210

[B14] Li T. Chen W. Liu F. Yao H. Huo Q. Zhang W.. (2023a). Benefits through innovative cropping patterns in the hilly regions of southwest China: an integrated assessment of emergy and economic returns. Agronomy 13, 2640. doi: 10.3390/agronomy13102640

[B15] Lin Y. Chen T. Liu S. Cai Y. Shi H. Zheng D.. (2022). Quick and accurate monitoring peanut seedlings emergence rate through UAV video and deep learning. Comput. Electron. Agric. 197, 106938. doi: 10.1016/j.compag.2022.106938

[B16] Lin H. Chen Z. Qiang Z. Tang S. Liu L. Pau G. (2023b). Automated counting of tobacco plants using multispectral UAV data. Agronomy 13, 2861. doi: 10.3390/agronomy13122861

[B17] Lin Y. C. Tong W. J. Chen D. R. Xu L. J. Jiang S. X. Wu Y. X.. (2023). Physiological response and its transcriptomic and secondary metabolic characteristics of tobacco seedlings under well-cellar style transplanting. J. Zhejiang Univ. (Agric. Life Sci.) 49, 305–318. doi: 10.3785/j.issn.1008-9209.2022.05.311

[B18] Ma J. Lu A. Chen C. Ma X. Ma Q. (2023f). YOLOv5-lotus an efficient object detection method for lotus seedpod in a natural environment. Comput. Electron. Agric. 206, 107635. doi: 10.1016/j.compag.2023.107635

[B19] Machefer M. Lemarchand F. Bonnefond V. Hitchins A. Sidiropoulos P. (2020). Mask r-CNN refitting strategy for plant counting and sizing in UAV imagery. Remote Sens. 12, 3015. doi: 10.3390/rs12183015

[B20] Rao X. Zhou L. Yang C. Liao S. Li X. Liu S. (2023). Counting cigar tobacco plants from UAV multispectral images via key points detection approach. Trans. Chin. Soc. Agric. Machinery 54, 266–273. doi: 10.6041/j.issn.1000-1298.2023.03.026

[B21] Tarolli P. Straffelini E. (2020). Agriculture in hilly and mountainous landscapes: threats, monitoring and sustainable management. Geogr. Sustain. 1, 70–76. doi: 10.1016/j.geosus.2020.03.003

[B22] Tong Z. Fang D. Chen X. Zeng J. Jiao J. Xiao J. (2020). Genetic analysis of six important yield-related traits in tobacco (Nicotiana tabacum L.). Acta Tabacaria Sin. 26, 72–81. doi: 10.16472/j.chinatobacco.2020.146

[B23] Veeramani B. Raymond J. W. Chanda P. (2018). DeepSort: deep convolutional networks for sorting haploid maize seeds. BMC Bioinf. 19, 289. doi: 10.1186/s12859-018-2267-2, PMID: 30367590 PMC6101072

[B24] Wang L. Xiang L. Tang L. Jiang H. (2021b). A convolutional neural network-based method for corn stand counting in the field. Sensors 21, 507. doi: 10.3390/s21020507, PMID: 33450839 PMC7828297

[B25] Wu D. Han W. Wang T. Dong X. Zhang X. Shen J. (2023). “Referring multi-object tracking,” in 2023 IEEE/CVF Conference on Computer Vision and Pattern Recognition (CVPR), Vancouver, BC, Canada. 14633–14642.

[B26] Wu C. Li Z. Yuan K. Wang D. Zhang F. Dai H.. (2022). Approach of full-mechanized production of tobacco under different ecological environments in hilly tobacco-growing areas. Modern Agric. Sci. 22, 125–130. doi: 10.3969/j.issn.1007-5739.2022.22.031

[B27] Wu S. Ma X. Jin Y. Yang J. Zhang W. Zhang H.. (2025). A novel method for detecting missing seedlings based on UAV images and rice transplanter operation information. Comput. Electron. Agric. 229, 109789. doi: 10.1016/j.compag.2024.109789

[B28] Wu Z. Sun X. Jiang H. Gao F. Li R. Fu L.. (2023). Twice matched fruit counting system: an automatic fruit counting pipeline in modern apple orchard using mutual and secondary matches. Biosyst. Eng. 234, 140–155. doi: 10.1016/j.biosystemseng.2023.09.005

[B29] Xie H. Fan Z. Li W. Rong Y. Xiao Y. Zhao L. (2016). “Tobacco plant recognizing and counting based on SVM,” in 2016 International Conference on Industrial Informatics - Computing Technology, Intelligent Technology, Industrial Information Integration (ICIICII), Wuhan, China. 109–113. doi: 10.1109/ICIICII.2016.0037

[B30] Xu G. W. Jian S. C. Song Y. M. Fang H. M. Qiu X. Y. Ming X. L. (2022). Design and experiment of cellar cavitation mechanism for crops of hilly mountains transplanter. Trans. Chin. Soc. Agric. Machinery 53, 105–113. doi: 10.6041/j.issn.1000-1298.2022.03.010

[B31] Ye X. Pan J. Shao F. Liu G. Lin J. Xu D.. (2024). Exploring the potential of visual tracking and counting for trees infected with pine wilt disease based on improved YOLOv5 and StrongSORT algorithm. Comput. Electron. Agric. 218, 108671. doi: 10.1016/j.compag.2024.108671

[B32] Zhang J. Tian M. Yang Z. Li J. Zhao L. (2024). An improved target detection method based on YOLOv5 in natural orchard environments. Comput. Electron. Agric. 219, 108780. doi: 10.1016/j.compag.2024.108780

[B33] Zhao B. Ding Y. Cai X. Xie J. Liao Q. Zhang J. (2017). Seedlings number identification of rape planter based on low altitude unmanned aerial vehicles remote sensing technology. Trans. Chin. Soc. Agric. Eng. (Transactions CSAE) 33, 115–123. doi: 10.11975/j.issn.1002-6819.2017.19.015

